# Disparities in Infectious Diseases among Women in Developed Countries^1^

**DOI:** 10.3201/eid1011.040624_08

**Published:** 2004-11

**Authors:** Camara Phyllis Jones, Tajudeen Ogbara, Paula Braveman

**Affiliations:** * Centers for Disease Control and Prevention, Atlanta, Georgia, USA;; † Orland Park, Illinois, USA;; ‡ University of California, San Francisco, California, USA

**Keywords:** infectious disease, woman, conference summary

## How Is Racism Operating Here?

To eliminate racial disparities in health, we need to examine the fundamental causes. The variable "race" is only a rough proxy for socioeconomic status or culture and is meaningless in terms of genes, but race is a precise marker for the social classification of persons in the United States' race-conscious society. Scientists now hypothesize that racism is a fundamental cause of health disparities between racial groups.

Racism can be defined as a system of structuring opportunity and assigning value based on the social interpretation of phenotype ("race"), and exists on three levels: institutionalized, personally mediated, and internalized. Institutionalized racism includes the structures, policies, practices, and norms that result in differential access to the goods, services, and opportunities of society by race. It includes the contemporary structural factors that perpetuate historical injustice, and creates the association between socioeconomic status and "race" observed in the United States. Personally mediated racism includes differential assumptions about the abilities, motives, and intents of others by race, as well as differential actions based on those assumptions. Internalized racism includes the acceptance by members of the stigmatized races of negative messages about their own abilities and intrinsic worth.

To deal with racism effectively, we must first name racism and put it on the agenda, monitoring for racial differences in exposures, opportunities, and outcomes. Then we must ask "How is racism operating here?" and identify mechanisms in structures, policies, practices, and norms, attending both to what exists and what is lacking. Finally, we must organize and strategize to act.

A six-question "Reactions to Race" module was piloted on the 2002 Behavioral Risk Factor Surveillance System by six states. It includes the questions "How do other people usually classify you in this country?" and "How often do you think about your race?" as well as questions on differential treatment at work and when seeking health care, and on emotional upset and physical symptoms because of race-based treatment.

## Women of Color and Infectious Disease Disparities in the United States

Racial and ethnic disparities exist with regard to certain types of infections common among women, including sexually transmitted diseases, urinary tract infections, pregnancy-related infectious diseases, and others (hepatitis E, hepatitis A, *Candida* vaginitis). Rates of gonorrhea, chlamydia, and HIV infection are higher among women of color than among white women. In 2002, of the total number of gonorrhea cases reported to the Centers for Disease Control and Prevention, 73% occurred in African-Americans. Black and Latino women account for ≈75% of all reported HIV infections among those 13–24 years of age in the United States, although they represent only ≈26% of the U.S. population.

Socioeconomic status is an important factor in these disparities. Substance abuse, sexual assault and abuse, employment, education, and poverty are just a few of the factors. Another contributing factor is the increase in sexually transmitted diseases among married African-American women because their husbands have sex with men. While antiinfective drugs are available for infectious diseases in women, perhaps the most important measure to reduce incidence is community-based education for both men and women. This is best accomplished by going to the community (schools, churches, barber shops, and other gathering places), rather than waiting for the community to come to you, and by increasing the number of accessible community health centers.

## Disparities in Infectious Diseases Affecting Women in Affluent Countries

There are disparities in infectious diseases by gender (for example, new HIV infections are increasing more rapidly among women than among men), but within affluent countries, disparities also exist between privileged and less-privileged groups of women. For example, within the United States, marked racial and ethnic differences exist in infectious disease death rates from pneumonia and influenza, septicemia, and HIV infection (the HIV death rate among black women is 13-fold higher than among white women). Marked socioeconomic differences in infectious disease mortality are also found, including striking differences in death rates from communicable diseases by educational attainment.

Health disparities in general are the domain of epidemiology, which is the science of the distribution of diseases and risk factors across populations. Yet, there is a firm basis in the fields of ethics (distributive justice) and international human rights (multiple principles, including nondiscrimination) for focusing on health disparities between more advantaged and less advantaged social groups. Health disparities that are of special concern are those that put already disadvantaged groups (for example the poor, disenfranchised racial and ethnic groups, and women) at further disadvantage with respect to their health.

Human rights and ethical considerations also lead us to have concern about disparities in the determinants of health. If we are formulating public health strategies on the basis of evidence, we must recognize the powerful evidence of the role of nonmedical as well as medical determinants of health disparities. Health professionals may focus on disparities in health care but should also support and be vocal advocates for eliminating disparities in some of the key social determinants of health, including education, early child development experiences, nutrition, and housing.

